# Case report: Gastric metastasis of breast cancer

**DOI:** 10.3389/fonc.2024.1430881

**Published:** 2024-09-27

**Authors:** Qiandi Zhao, De Zhang, Xinjian Wang

**Affiliations:** ^1^ School of Clinical Medicine, Shandong Second Medical University, Weifang, China; ^2^ Department of Gastrointestinal Surgery, Weihai Central Hospital, Weihai, China

**Keywords:** case report, breast cancer, gastric metastasis, retrospective analysis, immunohistochemistry

## Abstract

Breast cancer stands as the foremost malignant tumor among women globally, with postoperative recurrence and metastasis significantly impacting patient prognosis. While metastasis to various sites has been reported, gastric involvement remains uncommon. Presenting a case of gastric metastasis a decade post-breast cancer surgery, we underscore the rarity of this occurrence. Our patient, an elderly woman, underwent left breast modified radical surgery ten years prior, followed by adjuvant chemotherapy, maintaining favorable health until experiencing abdominal discomfort two months ago. Contrast-enhanced computed tomography (CT) of the chest and upper abdomen unveiled diffuse abnormal enhancement in the gastric body and sinus wall. Subsequent gastroscopy revealed an ulcer near the gastric antrum, with immunohistochemical staining confirming invasive lobular carcinoma metastasis from the breast. We further conducted an extensive review of 23 cases with detailed information retrieved from PubMed, elucidating clinicopathological, endoscopic features, diagnostic modalities, and contemporary treatment strategies for breast-stomach metastasis. Our findings underscore the imperative of regular postoperative surveillance for breast cancer patients. Timely detection, accurate diagnosis, and appropriate intervention are paramount in managing gastric metastasis, significantly influencing patient outcomes.

## Introduction

Breast cancer now ranks as the leading cancer among women globally, with the highest number of new cases diagnosed each year, the mortality rate of breast cancer stands at 6.9%, with postoperative recurrence and metastasis representing primary drivers of poor prognosis (1, 2). Complications related to tumor spread account for approximately 90% of patient deaths (3), with metastatic breast cancer rarely resulting in a cure (4). Common metastatic sites include local and distant lymph nodes, lungs, bones, liver, or brain, with gastrointestinal involvement, particularly in the stomach, being rare. Gastric metastasis from breast cancer occurs in only 0.3% of cases (5).

## Case report

A 78-year-old woman presented with a two-month history of epigastric pain and was admitted to our Gastroenterology Department. She had undergone modified radical surgery in September 2012 for left breast cancer, revealing invasive lobular carcinoma (2cm x 2cm) with metastases in the first (4/9), second (1/5), and third (3/3) lymph node stations. Immunohistochemistry showed positive estrogen and progesterone receptors, negative human epidermal growth factor receptor 2 and p53, and positive nm23 expression. Following surgery, she completed eight cycles of AC-T (cyclophosphamide + piroxicam + docetaxel) chemotherapy without adverse effects. The patient has managed coronary heart disease for 25 years and hypertension for 10 years with regular oral medications, achieving fair disease control.

**Figure 1:**
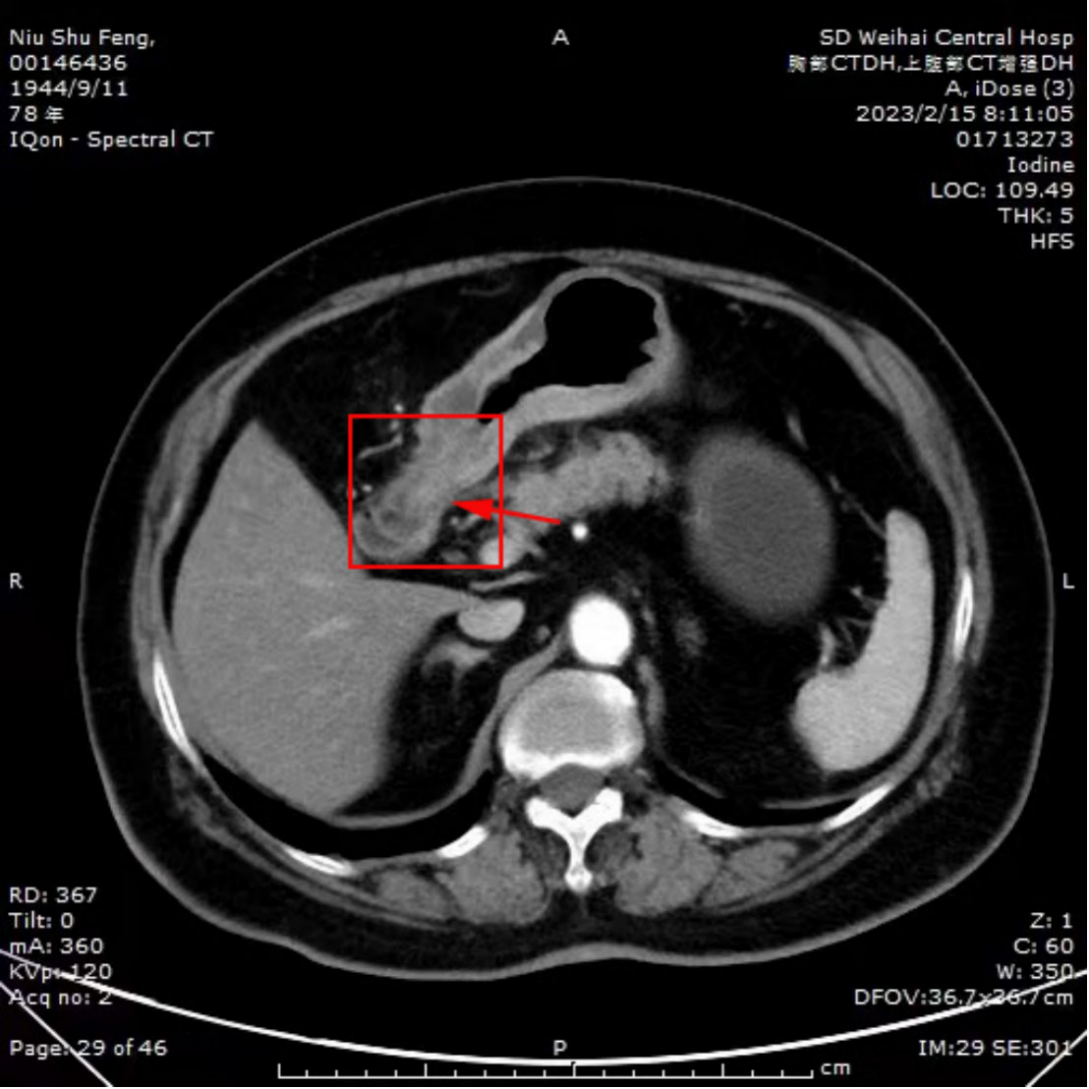
CT shows diffuse abnormal enhancement of gastric body and sinus wall.

**Figure 2:**
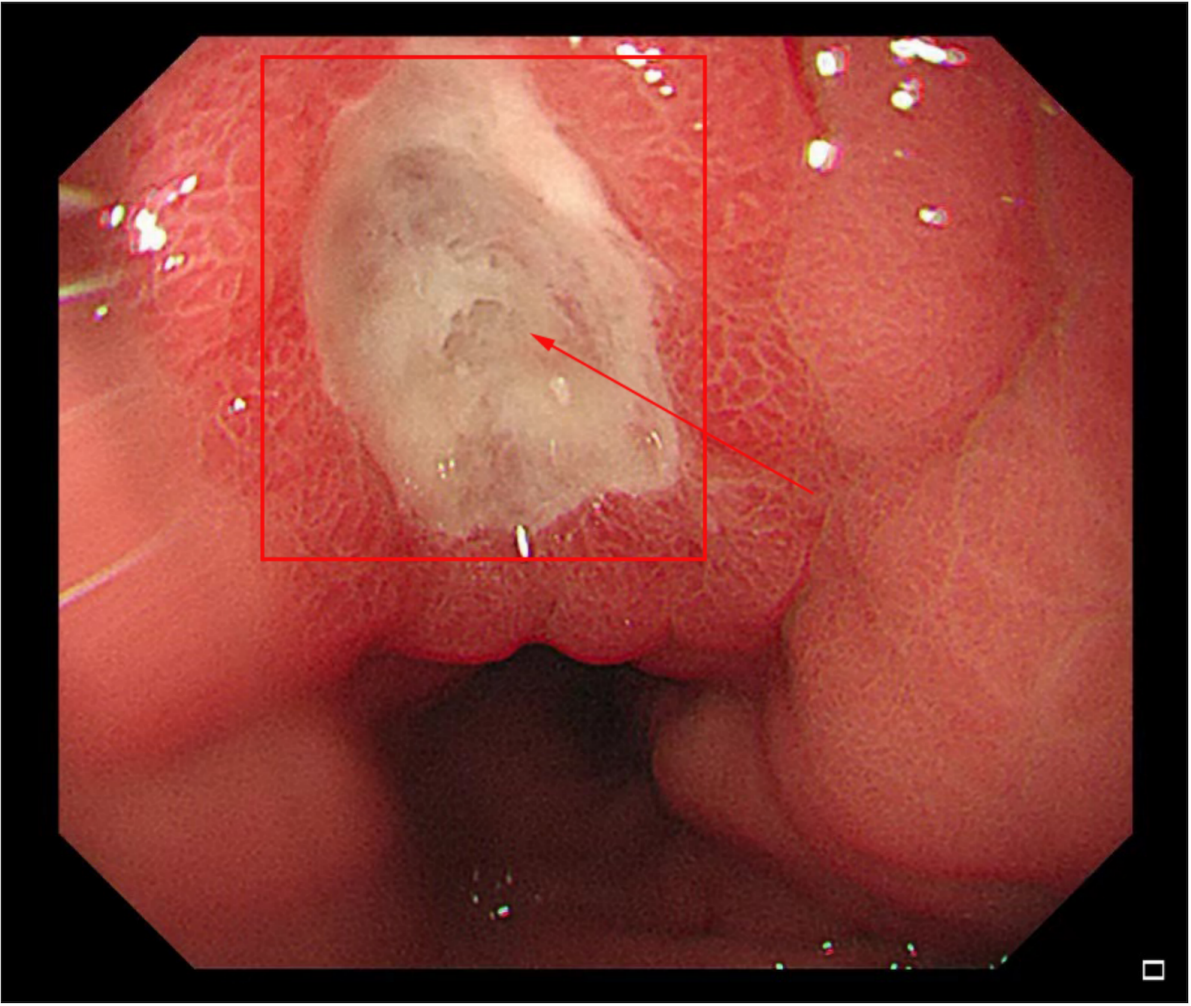
Gastroscope showed that the mucosa 4cm from the small curved mouth of the stomach was smooth, and the mucosa below 4cm to the anterior pyloric area was thick and stiff, with poor peristalsis. Mucosal congestion, edema, stiffness and contracture could be seen in the linear healing of gastric ulcer near the gastric angle. The mucosa of gastric antrum is thick and the entrance is deformed.

**Figure 3:**
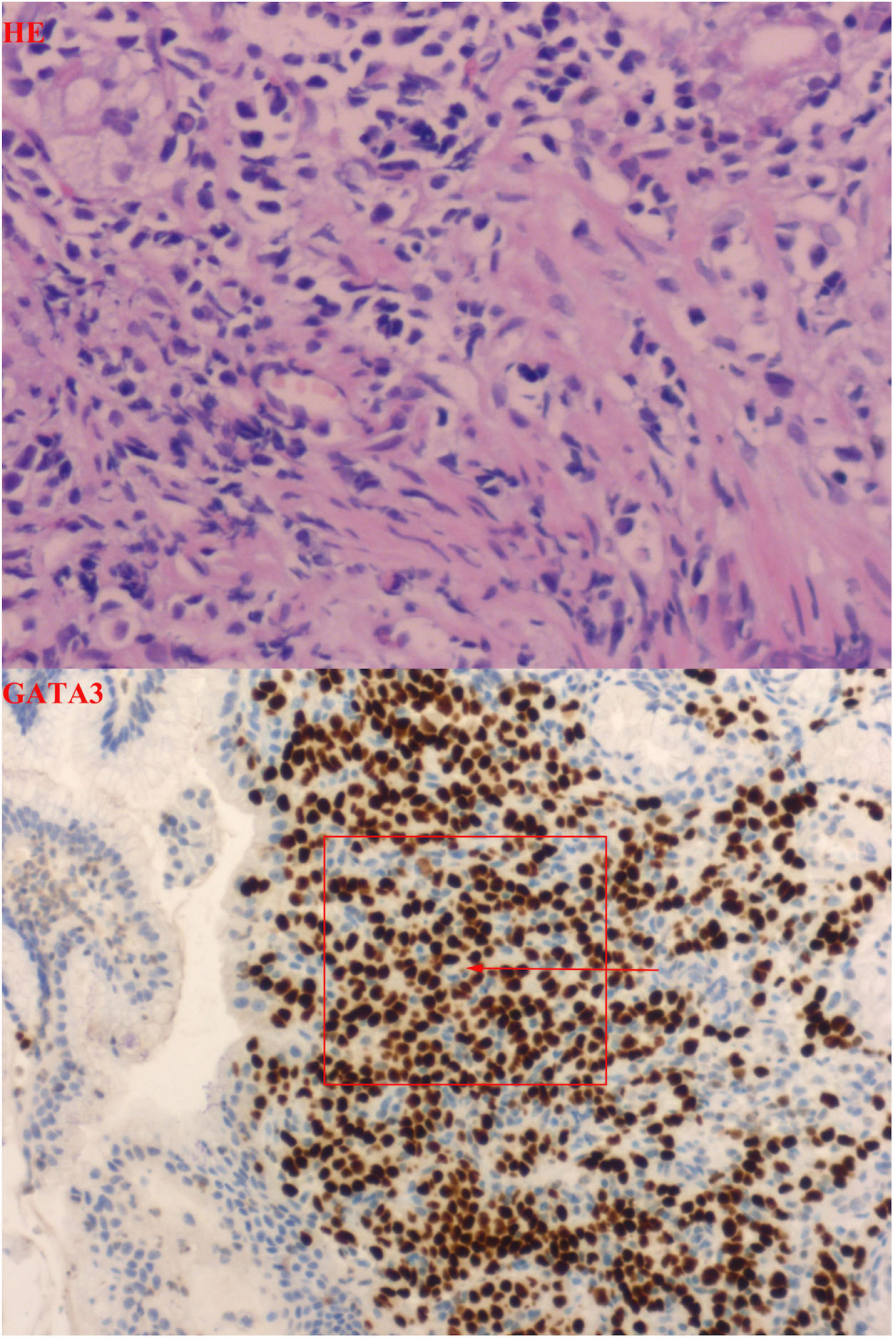
Pathology showed: HE staining pictures show that the normal histological structure of the stomach has been damaged, and we can see some heterosexual cells. Combined with the medical history, after adding immunohistochemical GATA3, it can be confirmed that these idioblast are from breast. Combined with Ki-67 index and E-cad, gastric metastasis of lobular carcinoma of breast was confirmed.

The patient presented with a two-month history of epigastric pain alongside markedly elevated serum CA72-4 levels upon hospital admission. Contrast-enhanced computed tomography of the chest and epigastric abdomen depicted diffuse abnormal enhancement of the gastric body and antrum wall (**Figure 1**). Gastric endoscopy revealed a smooth mucosa in the gastric fundus, while the middle part of the lesser curvature of the gastric body exhibited thickened and rigid mucosa. Notably, poor peristalsis was observed, accompanied by a visible 1.0x0.8cm ulcer near the gastric antrum corner. Surrounding mucosa displayed congestion, edema, rigidity, and contraction, with hypertrophy of the gastric antrum mucosa and deformity of the pyloric orifice. Severe chronic inflammation with interstitial fibroplasia and occasional small foci of mixed cells were evident in the gastric angle mucosa, while the anterior wall of the antrum part displayed chronic inflammation with occasional mixed cells in interstitial foci (**Figure 2**), consistent with metastatic invasive lobular carcinoma of the breast based on immunohistochemistry and patient history. Immunohistochemical staining showed CDX2(-), CK7(+), CK20(-), CK(+), Ki-67(+, 10%), Her2(1+), ER(3+), PR(+), GATA-3(+), and E-cadherin(-) (**Figure 3**). Following multidisciplinary team (MDT) discussion, the patient underwent transfer to the Department of Oncology for fulvestrant and perphenazine endocrine therapy. Subsequent to discharge, the patient has maintained regular drug treatment with complete resolution of abdominal pain symptoms and stable condition, undergoing scheduled follow-up.

## Discussion

The primary metastatic forms of breast cancer to the gastric region encompass invasive lobular carcinoma and ductal carcinoma. Invasive lobular carcinoma manifests a propensity for dissemination to various anatomical sites, including the gastrointestinal tract, gynecological organs, peritoneum, retroperitoneum, adrenal glands, and bone marrow. Conversely, invasive ductal carcinoma exhibits a predilection for metastasis to the lungs, bones, and liver (5). Importantly, gastric metastases frequently co-occur with metastases from other sites. In our reviewed literature, 15 patients were identified with simultaneous multi-organ metastases, constituting 65.2% of the total patient cohort (**Table 1**). Predominant metastatic sites included the bone, liver, and abdomen. However, a related investigation indicated no significant impact on survival associated with gastric metastasis from breast cancer, regardless of other organ involvement (6).

**Table 1:** The clinicopathological features of gastrointestinal metastases of breast carcinoma.

NO. of cases	23	Metastasis location (23/23)	
Age	63.26 ± 11.97 (39-85)	Esophagus	1 (4.35%)
Sex (23/23)		Stomach	23 (100%)
Male	0 (0%)	Duodenum	1 (4.35%)
Female	23 (100%)	Small bowel	0 (0%)
Type of breast cancer (23/23)		Appendix	0 (0%)
ILC	18 (78.26%)	Colon	1 (4.35%)
IDC	5 (21.74%)	Rectum	0 (0%)
Receptor of breast cancer		Interval time of metastasis (Y)(21/23)	6.53 ± 7.66 (-1.17-23)
ER (20/23)	18 (90.00%)	Receptor of GI metastasis	
PR (20/23)	16 (80.00%)	ER (13/23)	12 (92.31%)
HER2 (9/23)	1 (11.11%)	PR (12/23)	10 (83.33%)
Treatment of breast cancer (20/23)		HER2 (5/23)	1 (20.00%)
Operation (O)	17 (85.00%)	Treatment of GI metastasis (15/23)	
Conservative (C)	3 (15.00%)	Operation (O)	7 (46.67%)
AT after operation (13/23)		Chemotherapy (C)	11 (73.33%)
Chemotherapy (C)	12 (92.31%)	Endocrine therapy (E)	1 (6.67%)
Endocrine therapy (E)	1 (7.69%)	Radiation (R)	1 (6.67%)
Radiation (R)	1 (7.69%)	Time of follow-up (Y)(19/23)	8.72 ± 7.17 (1.33-24.25)

IDC, invasive ductal carcinoma; ILC, invasive lobular carcinoma; ER, estrogen receptor; PR, progesterone receptor; C, chemotherapy; E, endocrine therapy; O, operation; R, radiation; T, targeted; AT, adjuvant therapy; ND, not described; Y means year.

Breast cancer metastases to the stomach present with non-specific clinical symptoms akin to primary gastrointestinal tumors. Upon presentation, the patient exhibited upper abdominal pain resembling hunger pangs, alongside acid reflux and heartburn. Conventional imaging techniques, like upper gastrointestinal angiography or CT scans, typically indicate gastric wall thickening, prompting suspicion of metastasis. Gastroscopy stands as the primary modality for diagnosing gastrointestinal malignancies. Breast-stomach metastasis typically presents as ulcers, infiltrations, or masses observed in the gastric antrum and pylorus during endoscopic examination. Among the 16 patients reviewed in the literature, 11 exhibited diffuse infiltration (Bowman type IV), misdiagnosed as signet-ring cell carcinoma. Remarkably, all 11 patients had invasive lobular carcinoma as the primary breast cancer subtype. Moreover, invasion typically penetrates the submucosal and muscular layers, posing a challenge in distinguishing metastatic lesions from other tumors or benign conditions via surface biopsy alone, leading to diagnostic delays (10). Upon initial gastroscopy, the patient’s condition was initially diagnosed as chronic superficial gastritis and gastric ulcer due to the shallow nature of the biopsy. Subsequent to multidisciplinary team (MDT) deliberation, deeper biopsies coupled with immunohistochemistry revealed the true nature of the condition: gastric metastasis originating from breast cancer.

Immunohistochemical analysis stands as the foremost method in distinguishing primary and metastatic gastric cancer. A comprehensive panel comprising ER, PR, GCDFP-15, CK7, CK20, and CDX2 proves optimal in detecting gastric metastases originating from breast cancer. The estrogen receptor stands out as a highly sensitive marker for distinguishing metastatic breast cancer, albeit with limited specificity (7). GCDFP-15, predominantly found in gynecological tumors, external genitals, and salivary glands, demonstrates limited expression in gastrointestinal tumors, with a sensitivity range of 55-76% and specificity of 95-100% (8). Cytokeratin markers CK7 and CK20 aid in discriminating primary and metastatic gastric tumors, with CK7 prevalent in breast, lung, and ovarian adenocarcinomas, while CK20 shows heightened expression in stomach, colon, and pancreas (9). Additionally, E-cadherin serves as a pivotal marker in distinguishing lobular carcinoma from ductal carcinoma, showing a strong association with invasive lobular carcinoma metastasis. Approximately 90% of invasive lobular breast carcinomas exhibit E-cadherin deficiency, facilitating tumor metastasis, a pivotal determinant in breast cancer spread (10).

In our comprehensive analysis spanning 16 years, we found that a substantial majority (65.22%) of patients diagnosed with gastrointestinal metastasis of breast cancer were aged over 60 at the time of their initial breast cancer diagnosis. Remarkably, an overwhelming 78.26% of these patients were histopathologically identified as having invasive lobular carcinoma. These findings strongly suggest that elderly patients with invasive lobular carcinoma face a significantly heightened susceptibility to gastrointestinal metastasis of breast cancer. At present, the mode and mechanism of gastric metastasis of breast cancer are still unclear. Our findings reveal a median interval of 7 years between the initial diagnosis of breast cancer and the detection of gastric metastasis. Notably, this patient received a diagnosis after 10 years of postoperative chemotherapy. Our interpretation chemotherapy resistance and the dormancy of tumor stem cells as pivotal factors driving the prolonged metastasis and recurrence of breast cancer (11). Tumor stem cells undergo epithelial-mesenchymal transition (EMT), transforming into mesenchymal stem cells endowed with enhanced motility, invasiveness, and anti-apoptotic capabilities (12, 13). Furthermore, the concerted interplay among Helicobacter pylori, inflammatory cells, and chemokines within the gastrointestinal milieu fosters an environment conducive to tumor cell recruitment (14). At the same time, chronic inflammation may expedite EMT progression and carcinogenesis (15).

The primary treatments for primary gastric cancer typically involve surgery or chemoradiotherapy. However, a standardized treatment approach is lacking for patients with breast-gastric metastases. Our data revealed that over 70% of patients received hormone chemotherapy regimens, except for four cases requiring emergency surgery due to obstruction or perforation. Given that over 90% of patients with gastric metastases are estrogen receptor positive, we propose that a systemic therapy approach combined with hormone therapy represents the optimal treatment strategy for breast cancer patients with gastric metastases.

In conclusion, gastric metastasis originating from breast cancer represents a rare complication with clinically non-specific manifestations. Diagnosis primarily relies on gastroscopy biopsy and immunohistochemical staining. Presently, systemic therapy in conjunction with hormone therapy stands as the optimal treatment modality for patients with breast-stomach metastasis. Furthermore, this paper underscores the imperative of intensified postoperative surveillance for breast cancer patients, emphasizing the significance of timely detection, accurate diagnosis, and prompt intervention for individuals afflicted with breast-stomach metastasis.

## Data Availability

The original contributions presented in the study are included in the article/supplementary material. Further inquiries can be directed to the corresponding author.
